# Work-Related Burnout Among Physicians at Sultan Qaboos University Hospital: Prevalence and Risk Factors

**DOI:** 10.1192/j.eurpsy.2025.1796

**Published:** 2025-08-26

**Authors:** M. M. Albattashi, H. Mirza, S. A. Albusaidi

**Affiliations:** 1 psychiatry residency program, Oman Medical Specialty Board; 2 Department of behavioral medicine; 3 Department of medicine, Sultan Qaboos University Hospital, Muscat, Oman

## Abstract

**Introduction:**

Burnout is a work-related syndrome characterized by emotional exhaustion, depersonalization, and a reduced sense of personal accomplishment. It is particularly prevalent among physicians and it is associated with various personal and occupational risk factors. This study aims to assess the prevalence of burnout and its predictors among physicians across different specialties at Sultan Qaboos University Hospital (SQUH).

**Objectives:**

to assess the prevalence of burnout and its predictors among physicians across different specialties at Sultan Qaboos University Hospital (SQUH).

**Methods:**

A cross-sectional study was conducted from October 2023 to January 2024 involving Omani and non-Omani physicians from all specialties at SQUH, Muscat, Oman. Data were collected via an electronic and paper-based survey that included the Oldenburg Burnout Inventory and a sociodemographic questionnaire. Stepwise regression analysis was used to identify independent predictors of burnout.

**Results:**

A total of 353 physicians participated, yielding a response rate of 79%. The prevalence of burnout was 56.7% (n = 200). The majority of respondents were male 56.4%, with 88.4% being married and 66.3% having one to four children. Of the participants, 45% were consultants or senior consultants, and 70% had been practicing for over ten years. Independent predictors of burnout included male gender [OR 1.62 (1.01-2.59), p = 0.007], older age [OR 1.12 (0.66-1.89), p = 0.02], less than 6 hours of sleep per night [OR 2.57 (1.62-4.10), p < 0.001], administrative responsibilities [OR 2.75 (1.41-5.34), p = 0.015], and involvement in research activities [OR 3.75 (1.77-7.95), p = 0.036].

**Conclusions:**

The high prevalence of burnout among physicians at SQUH highlights the need for targeted interventions to mitigate burnout and its associated negative impacts. Addressing the identified risk factors is essential for promoting physician well-being and optimizing healthcare delivery.

**Disclosure of Interest:**

None Declared

